# Hospitals in South America

**Published:** 1919-03

**Authors:** 


### Hospitals in South America.

The hospital movement is extending to South America. That country has been behind most of the world in the matter of hospital facilities, there not being a single hospital in all the country under any American mission board. There is one union dispensary in Rio de Janeiro, but not even a nurses training school. The Methodist church proposes to use a part of the $80,000,000 it is now raising as a mission fund, to establish hospitals in South America where they are most needed.

